# Evaluating acceptability and experiences of pregnant women at high risk of developing gestational diabetes who take part in antenatal intervention trials: a qualitative systematic review

**DOI:** 10.1186/s12884-025-07854-z

**Published:** 2025-07-12

**Authors:** Gözde Sultan Çakır, Manar Abduljalil Bakhsh, Ola F. Quotah, Olivia Righton, Catherine V. George, Lucilla Poston, Sara L. White, Angela C. Flynn, Zoë Bell

**Affiliations:** 1 Department of Women and Children’s Health, School of Life Course and Population Sciences, King’s College London, London, UK; 2 Department of Nutritional Sciences, School of Life Course & Population Sciences, King’s College London, London, UK; 3 School of Population Health, Royal College of Surgeons in Ireland, Dublin, Ireland; 4 Department of Diabetes and Endocrinology, Guy’s and St Thomas’ NHS Foundation Trust, London, UK

**Keywords:** Gestational diabetes, Intervention, Pregnancy, Acceptability, Experience, Randomised controlled trials

## Abstract

**Background:**

Gestational diabetes mellitus (GDM) is associated with short- and longer-term adverse outcomes for both mother and child. The success of randomised controlled trials (RCTs) assessing interventions to prevent GDM depends in part on participant adherence to and acceptability of the intervention. A review of the nested-qualitative components of antenatal RCTs to prevent GDM is lacking. This qualitative systematic review aimed to evaluate the experiences of pregnant women at higher risk of developing GDM who took part in preconception and/or pregnancy interventions which aimed to reduce GDM.

**Methods:**

Electronic databases (MEDLINE, EMBASE, Cochrane Library), and reference and citation lists were searched up to February 2025. Studies were eligible if they included qualitative research methods to examine the experiences of pregnant women following an intervention to reduce GDM. We used the PRISMA (Preferred Reporting Items for Systematic Reviews and Meta-Analyses) framework, and the Critical Appraisal Skills Programme (CASP) qualitative checklist was used to assess the quality of the studies.

**Results:**

Of the 38,812 studies initially identified, 102 trials were screened for eligibility, and four met the inclusion criteria and were included. All were pilot RCTs using semi-structured interviews in high-income countries (UK *n* = 3, USA *n* = 1). Data were synthesised into three themes: (1) acceptability of the interventions, (2) adherence to the interventions, and (3) perceived change in knowledge, attitude and behaviour. Acceptability of interventions was influenced by awareness of GDM, extra support and antenatal care, and natural pharmacological supplements. Adherence to interventions was facilitated by the intervention content and delivery. Perceived change in knowledge, attitude and behaviour was facilitated by intervention specific components and perceived improvements in health.

**Discussion:**

This review identified factors influencing adherence and acceptability of interventions for pregnant women at high risk of GDM. It also highlighted a lack of embedded qualitative studies exploring women’s experiences of participating in antenatal interventions. The findings suggest that improving the design and implementation of pregnancy interventions requires greater attention to participants’ experiences and support systems. This study highlights the need for nested qualitative studies in RCTs to improve acceptability and adherence to pregnancy interventions.

**Supplementary Information:**

The online version contains supplementary material available at 10.1186/s12884-025-07854-z.

## Introduction

Gestational diabetes mellitus (GDM) is the most common complication during pregnancy, defined as hyperglycaemia with onset or first recognition during pregnancy [1]. GDM affects around 14% of pregnancies worldwide [2]and the prevalence has increased in parallel with the global rise in obesity [3]. There are short- and longer-term consequences for mothers and offspring from a pregnancy complicated by GDM. These include hypertensive disorders, preeclampsia and preterm delivery for the mother, and large-for-gestational age (LGA) and respiratory consequences for the child [2]. Longer term consequences include increased maternal risk of type 2 diabetes and offspring risk of child and adult obesity [4]. GDM is therefore a significant healthcare burden and novel preventative strategies are urgently required.

Due to the increased prevalence of GDM, randomised controlled trials (RCTs) have been conducted in an attempt to identify effective preventative interventions. A recent systematic review and meta-analysis (*n* = 84 RCTs) assessed the effectiveness of diet, physical activity (PA), nutritional supplement and pharmacological interventions to reduce GDM in women with known risk factors during preconception and in pregnancy [5]. Some of the component RCTs suggested that interventions of combined diet and PA, inositol, and vitamin D may reduce GDM; and that diet and PA were beneficial in women with two or more risk factors for GDM while inositol was effective in women with overweight or obesity [5]. In contrast, a recent systematic review (*n* = 128 RCTs) focused on evaluating the effect of trial quality on treatment effect estimates found that, with only studies at negligible risk of bias, no significant effect of combined diet and PA interventions on clinical outcomes, reporting only a reduction in gestational weight gain (GWG) of 1.10 kg (95% CI −1.62 to −0.58) [6].

A recent systematic review and meta-analysis (*n* = 41 RCTs) addressed the effectiveness of dietary and/or exercise interventions during pregnancy for preventing GDM in high-risk pregnant women [7]. RCTs that assessed the risk factor for overweight and/or obesity among participants as a total group, without body mass index (BMI) division and stratification of participants, were included. The meta-analysis findings showed a reduction in GDM for women who participated in the PA-alone intervention arm compared to standard care (OR 0.64, 95%CI 0.51, 0.80; p-value < 0.0001). Women who received dietary interventions during pregnancy were less likely to develop GDM than women who attended standard care. However, the meta-analysis findings were not significant (OR 0.73, 95%CI 0.51, 1.03; p-value 0.07) [7].

Although there is some evidence that interventions have possible benefits in reducing the risk of GDM in women with risk factors [5] the validity and transferability of the results of these RCTs in part depends on the participant’s adherence to and acceptability of the intervention components. Acceptability is a multi-dimensional construct that reflects how appropriate individuals delivering or receiving a healthcare intervention perceive it to be, based on their expected or experienced cognitive and emotional responses [8]. The Theoretical Framework of Acceptability (TFA) defines this construct through seven key components: affective attitude, burden, perceived effectiveness, ethicality, intervention coherence, opportunity costs, and self-efficacy [8]. Importantly, acceptability is a dynamic construct with a temporal dimension, which can be evaluated at different stages of an intervention: prior to exposure (conceptualisation and initial expectations), during implementation (ongoing experiences and anticipated continuation), and post-intervention (retrospective evaluation or projected feasibility in daily life).

One systematic review has reviewed the conduct and reporting of the acceptability, attitudes, beliefs and experiences of pregnant women who take part in antenatal diet and lifestyle interventions (*n* = 24 RCTs) [9]; this included RCTs with a nested qualitative component or any reporting of acceptability, attitudes, beliefs and experiences [9]. The review identified several factors that influence adherence to lifestyle interventions in pregnancy, including women’s beliefs, social support, and their perceptions of lifestyle changes [9]. This systematic review included all pregnant women with varying BMIs [9]. However, given the profound consequences of GDM and its growing burden on society, it is essential to focus on the experiences of pregnant women who are at high risk for developing GDM.

Understanding the acceptability and experiences of women participating in antenatal RCTs is a key element for trial effectiveness. This has not previously been examined in pregnant women with risk factors for GDM taking part in RCTs. Thus, this qualitative systematic review aimed to explore the experiences of pregnant women at higher risk of developing GDM who took part in interventions which aimed to reduce GDM before and during pregnancy.

## Methods

This review was part of a systematic review that aimed to evaluate the effect of interventions (behavioural, supplementation, and pharmacological) during the preconception period and/or pregnancy to reduce GDM in women who have been identified as being at higher risk for developing the condition; PROSPERO registration CRD42020177976. The present review focused on qualitative studies which examined the experiences of pregnant women following an intervention to reduce GDM and has been reported in accordance with the PRISMA guidelines [10] (Additional file 1, table A1).

The methods of Quotah et al., 2024 have been previously published [5]. In brief, three electronic databases (Medline, Embase and Cochrane Central Register of Controlled Trials) were first searched in February 2022 with a repeat updated search in February 2025 (Additional file 1, table A2, A3 and A4). Reference lists and citation searching using Google Scholar were conducted to identify additional relevant studies. Eligible RCTs were identified, including those included by Quotah et al., 2024 [5] as well as new studies identified during the updated search.

### Selection of studies and qualitative component evaluation

Records obtained from all databases were imported into the EndNote X9 reference management software [11] to exclude duplicate publications. These were subsequently imported to the screening management software Rayyan [12] to identify eligible studies. All titles and abstracts were screened in duplicate, independently by a review team (GSÇ, ZB, OFQ, MAB, OR). Full-text screening of eligible articles was done in duplicate and independently, with disagreements discussed and resolved by consensus opinion among three reviewers (GSÇ, ACF, ZB).

Inclusion and exclusion criteria were developed using PICOS criteria [13] (Additional file 1, table A5). For this qualitative systematic review, the only additional inclusion criterion beyond Quotah et al., 2024 was embedded qualitative data in RCTs. For inclusion, studies had to meet the following criteria: inclusion of qualitative research methods to examine the experiences of pregnant women following an intervention to reduce GDM. Qualitative research methods included for example, individual interviews with participants or focus groups. Studies were excluded if they utilised a survey without a qualitative component or other quantitative methodologies to examine participants’ experiences of interventions.

### Data extraction

The characteristics of included studies were extracted into standardised tables and checked for completeness and accuracy by three reviewers (GSÇ, ZB, ACF). As per PRISMA guidance, the data extraction template included: title, author, year, journal, specific aims, qualitative method, intervention characterisitics, study participant information, and study outcomes [10]. In relation to study outcomes, data on women’s experiences of intervention were extracted.

### Data analysis

A narrative synthesis approach was used to systematically review and synthesise findings from included studies [14]. This approach helps to develop a preliminary synthesis of findings of included studies and explore relationships in the data [14]. Details regarding intervention design were charted in narrative format with a descriptive paragraph created for each included study and undertaken by one author (GSÇ). Data immersion on experiences and acceptability was achieved through screening, reading and extraction phases. Included study findings were coded using an Excel spreadsheet adopting a thematic analysis approach [15]. Following this, codes were grouped to generate initial themes. Finally, the themes were visually mapped and inspected for overlap, and where necessary, refined through critical discussion by two authors (GSÇ, ZB) independently spreadsheet [15].

### Quality assessment

The Critical Appraisal Skills Programme (CASP) qualitative checklist was used to assess the quality of the studies [16] (Additional file 1, table A6). The checklist includes ten items to assess the relevance and reliability of the qualitative components of the RCTs. The quality of the studies was screened with ten questions. CASP criteria were applied to each study by three reviewers independently (GSÇ, ZB, ACF) and the categories ‘yes,’ ‘can’t tell,’ and ‘no’ were used to rate the domains. Any disagreements were recorded and resolved through discussion.

## Result

**Fig. 1:**
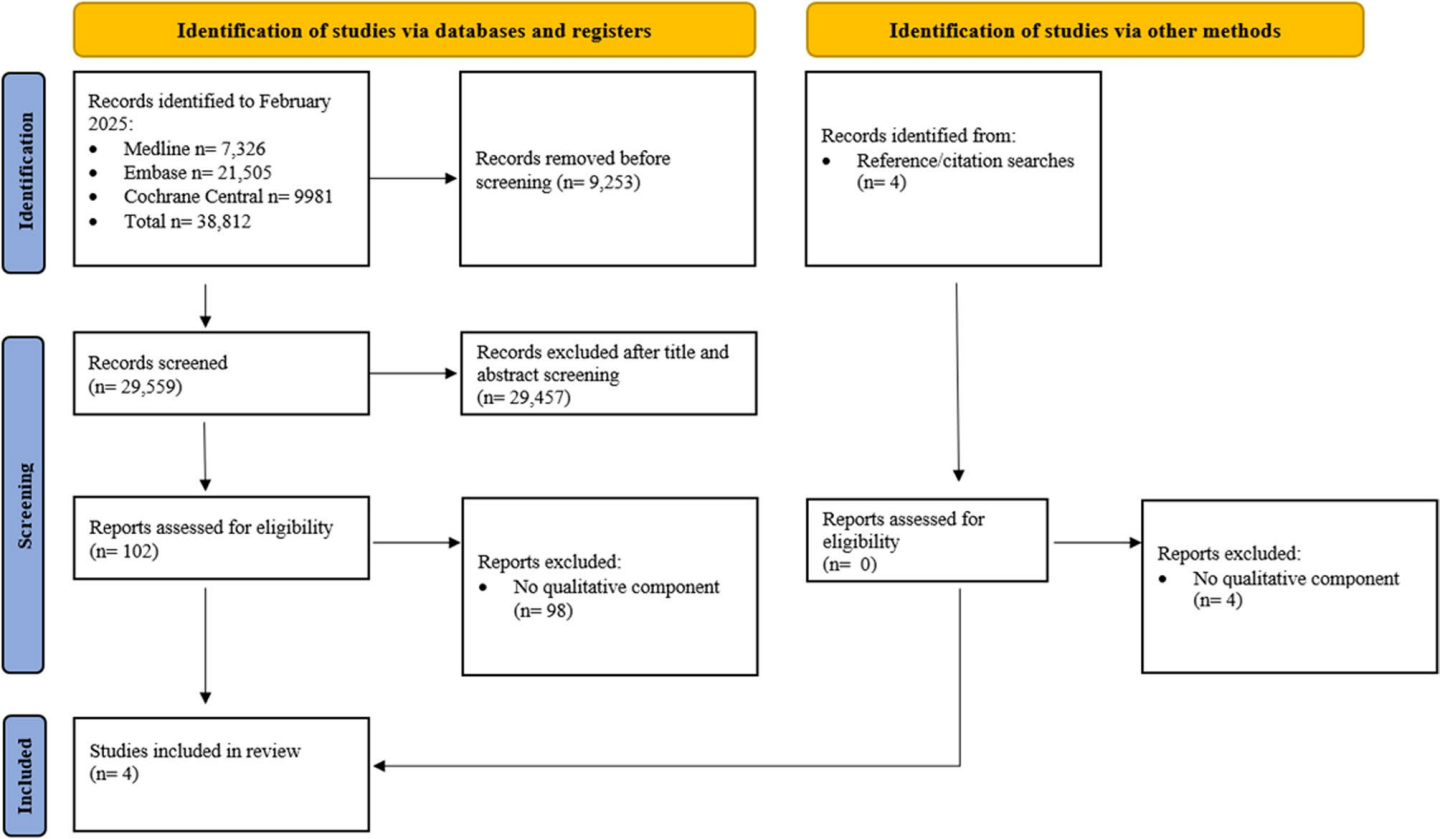
PRISMA flow diagram of searching and screening process

The study selection process is outlined in Fig. 1. A total of 38,812 articles were identified in the initial electronic database search, and four were identified from reference and citation searching. After screening titles and abstracts, 29,457 were excluded. 102 studies were full-text screened, and four reported an embedded qualitative component and were included in this review.

### Study characteristics and interventions

The characteristics of the included studies are summarised in additional file 1, Table A7. All studies were conducted in high-income countries: the United States of America (USA) [17] (*n* = 1) and the United Kingdom (UK) [18–20] (*n* = 3). All studies were pilot RCTs conducted in pregnant women living with overweight or obesity. Qualitative methods used by included studies were semi-structured interviews (*n* = 4) [17–20]. Participant sample size in the qualitative interviews ranged from 15 [20] to 21 [19]. Two studies reported the ethnicity of women interviewed; Amaefula et al. included women from Middle Eastern (*n* = 3), South Asian (*n* = 5), White European (*n* = 6), and Black African/Caribbean (*n* = 1) ethnic groups [20] while Poston et al. interviewed women from White (*n* = 8), Black (*n* = 12), and ‘Other’ (*n* = 1) ethnic groups [19] (see additional file 1, table A7). A single study reported a mean BMI of 37.6 amongst the interview sample [19] and education was only reported in one study [19].

Summary of interventions and reported outcomes are shown in additional file 1, table A8. Interventions included supplementation (Myo-inositol and folic acid [20]) and a behavioural approach (diet and physical activity [17, 19] diet only [18]). The supplementation intervention in Amaefule et al. utilised 2 g Myo-inositol and 200 µg folic acid, taken twice daily in powder form mixed with water until delivery [20]. Participants in this study were followed up with telephone consultations and mobile applications. Behavioural interventions (*n* = 3) were based on social cognitive theory [18–20] and hope theory [17] and ranged from dietary advice focusing on reducing carbohydrate intake [18] to combined interventions focusing on changing dietary intake and physical activity [17, 19]. Interventions were delivered in a variety of ways including remote use of paper-based or mobile applications [20]a combination of individual and group-based in-person sessions [19] one-to-one consultations with follow-up telephone contact [18] or web-based programs with online communications platforms [17]. Participants were provided with a range of tools to support the implementation of the intervention from a self-help booklet [18] or handbook [19] a pedometer [19] a DVD [17, 19] a logbook [17, 19] and a chart to record dietary goals and weight [18]. Acceptability was measured by all studies post-intervention, with one study also evaluating acceptability pre and during the intervention [20].

### Quality assessment

The quality assessment of the included studies is shown in additional file 1, table A6. All studies clearly stated the research aims for the qualitative component of their interventions. Methodology, research design, recruitment strategy, considered ethical issues, valuable research questions, and data collection methods were appropriate in all included studies. Some studies lacked reporting sufficient information in relation to data analysis, reflexivity and a clear statement of findings [17, 20].

### Thematic findings

This review identified three themes of acceptability and experiences of pregnant women at high risk of developing GDM who took part in antenatal interventions: (1) acceptability of the interventions (2) adherence to the interventions, (3) perceived change in knowledge, attitude and behaviour (Table 1).

**Table 1:** Synthesis of women’s experiences of antenatal interventions

Themes	Acceptability of the intervention			Adherence to the intervention		Perceived change in knowledge, attitude and behaviour
Subthemes	*Awareness of Developing GDM*	*Extra Support and Antenatal Care*	*Natural Pharmacological Supplement*	*The Intervention Content and Delivery*	*Intra-Inter Personal Relationships*	
Amaefule et al.,*2022*^*20*^	**X**	**X**	**X**	**X**	**X**	
Chang et al., 2023 ^17^		**X**		**X**	**X**	
Michalopolou et al.,2023 ^18^	**X**	**X**		**X**	**X**	**X**
Poston et al., 2013 ^19^	**X**	**X**		**X**	**X**	**X**

### Theme 1: Acceptability of interventions

The analysis identified different factors that influence the acceptability of the interventions.

#### Awareness of GDM

In three studies, participants described that perceived risk of developing GDM, previous experience with GDM, awareness of GDM risk factors such as a family history of diabetes, and concern about developing complications and weight gain in pregnancy positively influenced decisions to take part in and engage with the intervention [18–20].

*“Mostly because gestational diabetes was something I was a little bit concerned about. My dad is a diabetic and I’m obviously overweight*,* so I was just a little bit concerned about it. So*,* kind of any way to maybe avoid it would be of benefit*,* in my mind anyway.”* [18].

#### Extra support and antenatal care

In all studies, women described how the extra support and antenatal care they received before and throughout the intervention motivated them to participate and engage [17–20]. In one study assessing acceptability pre-intervention, women described how they felt they received adequate information about the study to make a decision about participating, and that they felt supported with their questions with their concerns addressed [20]. In three studies assessing acceptability post-intervention, participants described how they felt more cared for during pregnancy by intervention staff which made them willing and motivated to engage in the interventions [17–19].

*“You feel more looked after. You feel like it’s more private care*,* more special than just the normal midwife care or the GP. Because they just want to get you in and out really quickly. Cos*,* they have loads more people to see. But here you feel like you have the time to talk or say what you have to say. And ask any fears about anything*,* really*,* I think.”* [19].

#### Natural Pharmacological supplement

For the study which utilised a supplement, participants described feeling reassured that Myo-inositol was a ‘natural’ vegetarian composition. Most women part of this intervention stated that taking a natural supplement did not worry them, which motivated them to participate in [20].

*“it was something natural…a natural ingredient*,* nothing too chemical or new or anything. So it didn’t worry me”* [20].

### Theme 2: Adherence to the interventions

The analysis identified different factors that influence the adherence to the interventions.

#### The intervention content and delivery

Each study varied in their intervention content and delivery (Additional file 1, Table A8). In relation to intervention content, women in two studies positively reflected on the intervention content [18, 19]. Women from both these interventions appreciated the personalised and practical nature of the dietary advice, describing it as relevant and straightforward to apply [18, 19].

*“[…] from the beginning learning the study and being educated on the foods that will benefit you*,* how does it work*,* you know how it actually helps you in this situation has made a big difference.”* [18].

In two studies, women reported specific dislikes regarding the intervention content, which appeared to negatively influence adherence [17, 20]. Ameafula et al.,2022 noted that the powder formulation of Myo-inositol, alongside its perceived exacerbation of pregnancy-related nausea, contributed to reduced adherence [20]. Whereas Chang et al.,2023 found that participants expressed dissatisfaction with the web-based component of the intervention, particularly due to its written text and repetitive format, which was perceived as monotonous and led to declining engagement over time [17]. Furthermore, the rigid structure of weekly assigned topics was considered misaligned with participants’ immediate needs, such as receiving dietary advice during emotional stress.

In relation to intervention delivery, one study provided a supplement in the form of Myo-inositol [20]. Participants retrospectively suggested a preference for using a tablet rather than powder form in future studies. An additional challenge to adherence for these women included the restrictions of taking the supplement twice daily, 1 h before or after a meal [20]. Further, despite it being a natural supplement there were perceived side effects. In women who experienced prolonged nausea and vomiting during pregnancy, they discontinued the supplements when they felt that these symptoms were worsened by taking the supplement which affected adherence [20].

“*…if it was in tablet form it would be easier for them to just swallow it*,* just like the Pregnacare… But because it’s in liquid they have to take a mouthful and mouthful and mouthful… And the main reason for the withdrawal again is the fact that they were having very bad morning sickness.”* [20].

Two studies reported using group-based sessions to deliver dietary advice and/or physical activity [18, 19]. In two studies, women reported enjoying having smaller group diet sessions which provided more depth and opportunities to raise questions and discuss each other’s experiences, and created a more tailored approach which was appropriate for them [18, 19].

“*I thought it was going to be healthy eating and exercising. I thought it was going to be like how they tell you in the news*,* that we have to eat better. Or what you hear media- wise. But it’s more in-depth and more suitable to how you are*,* basically. It’s more fitted to how you are. Instead of every thousand people. It’s just for you. It’s more suitable that way*,* I feel.”* [19].

Two studies used technology-based delivery for part of their intervention [17, 20]. In one study, the delivery of intervention content via the web was neither preferable nor dissatisfactory to women; rather, they had concerns regarding the length and format of activities in the intervention [17]. In another study, a mobile app was incorporated into the intervention to support adherence through features such as automated reminders and digital tracking [17, 20]. Among the few participants who were able to download the app, it was perceived as helpful in maintaining adherence to the supplement regimen [20]. However, technical issues, particularly limited internet connectivity at the point of randomisation, prevented many participants from accessing the app [20]. Some women who could not use the app expressed that they might have adhered more consistently if they had received digital reminders and had been able to track their intake using their personal phones, rather than using a physical diary [20].

*“… I think an App or something would have been better… something that reminded me to take it… I just saw the booklet*,* it’s just like an admin thing. That didn’t help me. So an App might have done because I’ve got all the pregnancy Apps on my phone to remind me to take other things ….”* [20].

All studies providing informaiton within written materials found that the handbook/self-booklet and pre-written goals (web module) were generally well received. Behavioural strategies like setting and reflecting on weekly goals, self-monitoring, small, gradual changes and dietary advice were included as enablers to adherence of interventions [17–19].

*“Really*,* really useful [the dietary consultation]. I felt like sometimes that you go into pregnancy– especially with my first pregnancy – and no one advises you not to eat for two […] This time it was like you’re having the right guidance of - this is what you should really eat and shouldn’t eat.”* [18].

Some participants described feeling more motivated to engage in physical activity, noting that using a pedometer and tracking their steps encouraged them to aim for daily targets [19].

“*If I didn’t leave home at all [one day]*,* I would have like 1000 steps and I’m like “Oh my God*,* that’s really [low].” it sort of motivated me to try and do something about it the next day. …if I hadn’t done this [research]*,* I suppose I’d still be in that mindset*,* that*,* “Oh*,* I am pregnant*,* I’m not allowed to do anything” whereas now*,* because of having looked at my step count*,* I am very aware that I have to stay active and when I don’t*,* it does bug me.”* [19].

#### Intra-Inter personal relationships

All studies delivered support through staff specifically trained to provide tailored health messages to participants [17–20]. In one study, participants valued the personalised approach of the health coach [17]. This personalised guidance was reported to increase motivation and confidence [17]. Similarly, in other studies, women described the research staff as approachable and informative [18–20].

*“It’s [the advice] very well explained in the book*,* and you’ve been brilliant as well*,* sort of explaining everything. So*,* I’ve always felt very well informed with everything. So*,* it’s been easy to follow. For me anyway*,* it just seems quite effortless*,* really*,* because you’ve made it so easy.* ” [18].

In three of the included studies, social support from partners and family members was described as an important facilitator of adherence to interventions. While partners and family members were not directly involved in the interventions, women frequently mentioned the emotional and practical support they received from them as beneficial in sustaining healthy behaviours [18–20].

*“My partner kind of joined me because he needs to lose weight. Like all of us in the family did it*,* rather than just me [.] It was like a household change*,* so that’s why I think it wasn’t as hard to implement or as difficult […] Even like my mum and dad*,* they were really keen to read all the information and take things away from that because they felt like they could make their diet healthier.”* [18].

A range of barriers were noted to affect acceptability and thus adherence to interventions [17, 18].

These included factors such as competing priorities, school or work time and physical symptoms of pregnancy, such as pelvic pain [18, 19]. In one web-based study, participants retrospectively described how weekly intervention sessions created additional burdens on daily life, and women suggested reducing its frequency [17]. Similarly, in an intervention consisting of 8 weekly group sessions delivered by a health trainer, women reflected that although they valued the group approach, commitment was variable due to competing priorities [19].

“*I also found it quite hard saying ‘Oh*,* I need eight Thursday afternoons off work’*,* and I just felt like I was taking advantage of them by taking extra time off work.”* [19].

Another barrier that participants reported was participants’ perceived body image. In one study, which delivered a behavioural intervention using a combination of a paper-based handbook/self-booklet, some women described how their long-standing experiences with dieting and negative emotions around weight influenced their engagement with the intervention [18]. While most participants found self-monitoring and self-weighing helpful for maintaining healthy behaviours, a few expressed discomfort due to body image concerns [18].

*“[…] I think it’s just from being overweight my whole life so.always sort of getting on the scale and logging the number and then try to sort of come to terms with that in my last pregnancy. Because I knew it was ok to gain weight*,* it was natural and normal. But it’s still sort of like a bit of a mental barrier*,* to be like ok I know I’m going to weigh more this time*,* I’m currently at the heaviest that I’ve ever been. But I know that’s fine because I’m incredibly pregnant. It’s just.as soon as you see that thing on your head. on the scale*,* it’s kind of sad I guess.”* [18].

#### Theme 3: Perceived change in knowledge, attitude and behaviour

Two studies evaluated whether women’s participation in an intervention led to a perceived change in their knowledge and attitude towards a healthy lifestyle, their eating and/or physical activity habits, stress and emotion management [18, 19]. Most participants were receptive to the dietary advice provided by the health trainer/research dietitian and spoke of their intention to keep the dietary changes beyond pregnancy.

*“I can keep those changes for the long term and it won’t be so difficult for me to lose the weight after the birth*,* so that’s another that compared to my first pregnancy I was kind of more lost on what to do and because I’ve been already doing the changes and sticking to them for weeks*,* or months*,* I know I can keep it.”* [18].

Participants mentioned they had started swapping with healthier food options for rice, potatoes, and other starchy foods [19].

“*Instead of the basmati rice*,* I’d had the normal long grain rice*,* and instead of mashed potato*,* you can have sweet potatoes…so changing that*,* swapping… The benefits are you’re definitely not gaining that much weight*,* which is a plus”* [19].

The intervention supporting making small, gradual behaviour changes was also emphasised that making small, gradual changes increased the acceptability of interventions [18].

*“you did suggest just trying to change one or two things at a time*,* so it didn’t feel completely drastic either […]”* [18].

Participants’ families were also starting to adopt healthy eating with making small gradual dietary changes [18].

*“[…] my husband has noticed this difference as well. We just both feel a bit healthier. It’s been a good effect on the family.”* [18].

Most participants who took part in the two studies stated that the knowledge gained during the intervention positively influenced their attitudes and behaviours toward adopting a healthier lifestyle after pregnancy [18, 19]. This directly links to the heightened sense of health. For some, this was accompanied by a heightened sense of being observed and evaluated, which in turn led to feelings of guilt when they perceived themselves as failing to meet the expectations of the programme. These feelings were often linked to concerns about doing what was best for their baby, and to internal pressures to conform to a healthy ideal [18, 19].

*“…quite bad*,* and I felt that … that I wasn’t doing good … I wasn’t doing what was good for my baby … by not being healthy and fit and … and all of that*,* I felt like I was doing something wrong*,* so … I don’t know…you’re on a diet of guilt*,* you know*,* you should be eating this because otherwise you’re doing badly.”* [19].

However, participants also noted that they felt it would be difficult to make changes if the motivation to protect the baby’s health disappeared [18].

“*I want to start on my diet after my baby’s born. More healthy cooking and stuff. And once or twice a week*,* swimming and stuff like that. It makes you feel positive about yourself to do more. So afterwards you feel*,* okay*,* if I can do this while I’m pregnant*,* I can do 100 times more when I’m not. So I think it’s a motivation thing. It makes you think about*,* basically*,* it makes you think about your health during your pregnancy.”* [19].

## Discussion

This review sought to explore the experiences of women identified as higher risk of GDM who took part in antenatal interventions designed to reduce GDM. This is one of the first qualitative systematic reviews aimed at addressing this gap in the literature. Only four interventions were identified, each with an embedded qualitative component from which synthesis was conducted. The identified studies highlight numerous factors that affected the adherence and acceptability of interventions designed to prevent GDM. This review draws attention to the importance of including qualitative components in antenatal intervention studies to help improve understanding of the experiences of interventions and thus improve the acceptability and implementation of future interventions.

The findings of this systematic review highlight that nesting qualitative methodologies within interventions add in-depth insights into the factors affecting acceptability of interventions. Firstly, across the studies it was evident that the extra support and antenatal care provided as part of interventions made participants feel more cared for, thereby improving intervention acceptability. Pregnancy is a time when mothers can feel a heightened sense of anxiety and worry over pregnancy outcomes; this has been shown amongst women at risk of GDM [21]. Taking part in interventions offers a greater number of touchpoints for women with healthcare professionals or trained health advisor to ask questions about GDM and pregnancy. Maintaining multiple contact points with healthcare professionals as part of an intervention is an important component to consider for future studies [22].

An emotional dimension that emerged was the experience of guilt among some women participating in antenatal lifestyle interventions. Guilt was felt by women when they had not met dietary or physical activity expectations set by the intervention, or felt they might be compromising their baby’s health due to personal limitations or non-adherence. This highlights the importance of providing behaviour change support in a way that is both empathetic and empowering [22]especially given that women with heightened risk of GDM, already experience elevated levels of pregnancy-related anxiety [23]. Acknowledging and addressing guilt as part of the intervention framework may be an approach for future intervetions to enhance acceptability as well as support long-term behaviour change in pregnant women at risk of GDM.

This systematic review shows that heightened awareness of GDM and the risks associated with the condition motivated women to participate and adhere to antenatal interventions aimed at supporting them to adopt a healthier lifestyle for their babies and their health after pregnancy. This finding supports the wider literature that health knowledge is an important factor to behaviour change. A systematic review and meta-synthesis (*n* = 92) which aimed to identify factors influencing women’s health behaviour during pregnancy found that women’s general awareness of the importance of behaviour change acted as a facilitator, while limited understanding of specific health outcomes was a barrier to adopting and sustaining those changes [24]. Many women may only become aware of their risk for GDM during pregnancy and not have prior experience or knowledge of the condition [24].

Furthermore, our findings reinforce the preference for personalised approaches and the use of positive language when delivering intervention messages. This aligns with evidence from preconception care studies targeting women of childbearing potential, where such approaches were associated with improved knowledge and behaviour change [25]. These strategies appear to enhance acceptability and engagement, particularly when individuals feel that the advice is relevant, supportive, and sensitive to their personal circumstances [25, 26]. Therefore, integrating positively framed, personalised messaging may therefore be a crucial component in increasing the success of antenatal interventions targeting high-risk pregnant women.

While some included studies used technology-based interventions such as mobile apps and web modules, our review found limited evidence to draw firm conclusions about their effectiveness or acceptability for educational or behavioural change among women of childbearing potential. In line with this, the current evidence remains limited to draw definitive conclusions [27]. These findings highlight the need for further research to evaluate the long-term effectiveness, acceptability, and equity of digital health interventions in antenatal care.

This review found that adherence to interventions and in particular, behaviour change required social support from partners or family. This finding adds to the wider body of evidence showing the importance of partner’s roles in antenatal interventions. A qualitative review investigating women’s experiences of social support during pregnancy found that women needed emotional support, especially from their partners to motivate behaviour change [22]. Engaging partners as part of the lifestyle change during the antenatal period has also been identified as a key facilitator to behaviour change [23]. However, it is important to recognise that partner involvement may also act as a barrier for some women, particularly when the perceived mental load of ensuring their partner’s compliance with dietary or behavioural changes adds additional stress, as highlighted by recent research [28]. Thus, future interventions should consider pre-intervention qualitative research to assess the acceptability of including partners to take part in some of the intervention components as a mechanisms to further increase the effectiveness of interventions and sustain behavioural changes.

This review found that adherence to interventions was affected by a range of reported competing logistical issues [17–20] such as time constraints from work and caring responsibilities. Although intervention design and components varied across the included studies it is an important finding highlighting the need for flexible intervention delivery and the need to include patient representations from the start of the research cycle in shaping study design [29]. Previous reviews exploring pregnant women’s acceptability, attitudes, and beliefs in diet and lifestyle interventions support this finding with travel considerations and life events identified as the two biggest barriers to intervention adherence [9]. Crucially, assessing acceptability should not be limited to the pre-intervention stage; instead, it should be evaluated at multiple time points, before, during, and after the intervention, to better understand evolving barriers and support needs throughout the intervention journey [6].

This review also explores how the concept of acceptability has been conceptualised and investigated in antenatal interventions targeting women at high risk of developing GDM. In line with the dimensions proposed by the Theoretical Framework of Acceptability (TFA) [8]women’s accounts frequently reflected elements such as affective attitude, perceived effectiveness, intervention coherence, opportunity cost, self-efficacy and burden, particularly through their appreciation of personalised support, the clarity of intervention messages, and the practicality of dietary or physical activity guidance. However, dimensions were rarely addressed explicitly, suggesting a gap in understanding how women assess the moral alignment of interventions or their own confidence in engaging with them. Notably, only one study [20] assessed acceptability both before and after the intervention; the remaining studies evaluated it solely post-intervention [17–19]. This limits insights into how initial expectations may evolve over time and whether perceptions of acceptability shift as women navigate the intervention alongside the demands of pregnancy. Future research should therefore consider a more comprehensive and longitudinal approach to evaluating acceptability, to better inform both the timing and content of assessments [29, 30].

### Strengths and limitations

To the best of our knowledge, this is the first systematic review exploring the qualitative components of interventions in pregnant women at high risk of developing GDM. Strengths of this review include the a priori registration on PROSPERO, use of a PICOS framework to develop a rigorous search strategy with well-defined eligibility criteria [31]quality appraisal on included studies and reporting using PRISMA guidelines. In recognition of the limitations of database searches, reference and citation screening was included as part of a comprehensive search strategy.

However, this review has several limitations. Only four studies from two high-income countries with different healthcare systems were included, thus the transferability of the findings is limited. Moreover, differences in intervention design across the included studies constrained the ability to conduct a more structured comparison and make transferable conclusions for future antenatal interventions. A key limitation inherent to qualitative evidence synthesis is that the findings are not generalisable in the same way as quantitative results.

Additionally, methodological issues within the included studies may have influenced the findings. In some cases, the same individual was responsible for both delivering the intervention and conducting the interviews. This dual role may have introduced social desirability bias, whereby participants may have felt inclined to emphasise positive experiences or minimise challenges, potentially affecting the authenticity of their accounts. Such dynamics underline the importance of reflexivity and researcher positionality in qualitative health research. Moreover, limited information was often provided on interview training, analytic reflexivity, and steps taken to mitigate bias, which complicates assessments of rigour across studies. Furthermore, the studies included in this review were restricted to English-language publications, which may have excluded relevant studies published in other languages. However, no studies were excluded for this reason during the screening process.

### Implications

Although numerous RCTs have been conducted with pregnant women at high risk of developing GDM, only four studies incorporated qualitative components. This highlights the importance of embedding qualitative research within trials to capture the lived experiences of participants, which can significantly improve intervention acceptability beyond quantitative evaluation. Future research should explore ways to reduce the burden of interventions on participants. This could include adapting the timing of sessions to accommodate childcare and employment responsibilities, as well as utilising technological solutions to minimise the need for in-person visits, reducing the travel burden for participants.

A definition of acceptability and its constituent domains is now available. Future research should adopt this definition and apply this framework to assess acceptability in healthcare antenatal interventions. Additionally, future trials should use standardised frameworks and core outcome sets to improve consistency in how interventions are designed and evaluated. This would help researchers compare results more easily across studies. At the same time, some flexibility is needed to adapt interventions to different cultural and local contexts.

## Conclusion

This qualitative systematic review found that, despite the publication of many RCTs investigating the prevention of GDM in high-risk pregnant women, there is a lack of embedded qualitative studies exploring women’s acceptability and experiences of antenatal interventions. Future research must adopt a definition of acceptability and a more integrated approach by incorporating qualitative methods alongside intervention trials to better understand the factors influencing women’s acceptability and adherence. Addressing these gaps is essential to designing more effective interventions in the future.

## Supplementary Information

Supplementary Material 1: Table A1 PRISMA guidelines. Table A2 Literature search strategy (MEDLINE 1946 to February, 2025). Table A3 Literature search strategy (EMBASE 1946 to February, 2025). Table A4 Literature search strategy (Cochrane to February, 2025). Table A5 Summary of PICOS criteria for the inclusion of studies. Table A6 Critical Appraisal Skills Programme (CASP) qualitative checklist table. Table A7 Included study characteristics. Table A8 Summary of interventions and reported outcomes.

## Data Availability

No datasets were generated or analysed during the current study.

## Abbreviations

BMI: Body Mass Index
CASP: Critical Appraisal Skills Programme
GDM: Gestational Diabetes Mellitus
GWF: Gestational Weight Gain
GI: Glycemic Index
LGA: Large-for-Gestational-Age
NICE: National Institute for Health and Care Excellence
NIHR: United Kingdom National Institute of Health Research
RCTs: Randomised Controlled Trials
PCOS: Polycystic Ovary Syndrome
PA: Physical Activity
PRISMA: Preferred Reporting Items for Systematic Reviews and Meta-Analyses
UK: United Kingdom
USA: United States of America
