# Supplementary material for: Evaluating acceptability and experiences of pregnant women at high risk of developing gestational diabetes who take part in antenatal intervention trials: a qualitative systematic review
Source: BMC Pregnancy Childbirth. 2025 Jul 12;25:754. doi: 10.1186/s12884-025-07854-z (PMC12255016; doi:10.1186/s12884-025-07854-z)
Supplement: Supplementary file 1 — Supplementary Material 1: Table A1 PRISMA guidelines. Table A2 Literature search strategy (MEDLINE 1946 to February, 2025). Table A3 Literature search strategy (EMBASE 1946 to February, 2025). Table A4 Literature search strategy (Cochrane to February, 2025). Table A5 Summary of PICOS criteria for the inclusion of studies. Table A6 Critical Appraisal Skills Programme (CASP) qualitative checklist table. Table A7 Included study characteristics. Table A8 Summary of interventions and reported outcomes. [file 12884_2025_7854_MOESM1_ESM.docx]

**Evaluating acceptability and experiences of pregnant women at high risk of developing gestational diabetes who take part in antenatal intervention trials: a qualitative systematic review**

**Additional file 1**

**Table A1** PRISMA guidelines

**Table A2** Literature search strategy **(**MEDLINE 1946 to February 2025)

**Table A3** Literature search strategy **(**EMBASE 1946 to February 2025)

**Table A4** Literature search strategy (Cochrane to February 2025)

**Table A5** Summary of PICOS criteria for the inclusion of studies

**Table A6** Critical Appraisal Skills Programme (CASP) qualitative checklist table

**Table A7** Included study characteristics

**Table A8** Summary of interventions and reported outcomes

**Table A1** PRISMA guidelines

| **Section/topic** | **#** | **Checklist item** | **Reported on page #** |
| --- | --- | --- | --- |
| **TITLE** | | |  |
| Title | 1 | Evaluating acceptability and experiences of pregnant women at high risk of developing gestational diabetes who take part in antenatal intervention trials: a systematic review | Title page |
| **ABSTRACT** | | |  |
| Structured summary | 2 | Provide a structured summary including, as applicable: background; objectives; data sources; study eligibility criteria, participants, and interventions; study appraisal and synthesis methods; results; limitations; conclusions and implications of key findings; systematic review registration number. | 2-3 |
| **INTRODUCTION** | | |  |
| Rationale | 3 | Describe the rationale for the review in the context of what is already known. | 4 |
| Objectives | 4 | Provide an explicit statement of questions being addressed with reference to participants, interventions, comparisons, outcomes, and study design (PICOS). | 6 |
| **METHODS** | | |  |
| Protocol and registration | 5 | Indicate if a review protocol exists, if and where it can be accessed (e.g., Web address), and, if available, provide registration information including registration number. | 6 |
| Eligibility criteria | 6 | Specify study characteristics (e.g., PICOS, length of follow-up) and report characteristics (e.g., years considered, language, publication status) used as criteria for eligibility, giving rationale. | Page 6 and Additional file 1 /Table A5 |
| Information sources | 7 | Describe all information sources (e.g., databases with dates of coverage, contact with study authors to identify additional studies) in the search and date last searched. | 7 |
| Search | 8 | Present full electronic search strategy for at least one database, including any limits used, such that it could be repeated. | 7 |
| Study selection | 9 | State the process for selecting studies (i.e., screening, eligibility, included in systematic review, and, if applicable, included in the meta-analysis). | 7 |
| Data collection process | 10 | Describe method of data extraction from reports (e.g., piloted forms, independently, in duplicate) and any processes for obtaining and confirming data from investigators. | 8 |
| Data items | 11 | List and define all variables for which data were sought (e.g., PICOS, funding sources) and any assumptions and simplifications made. | 7-8 |
| Risk of bias in individual studies | 12 | Describe methods used for assessing risk of bias of individual studies (including specification of whether this was done at the study or outcome level), and how this information is to be used in any data synthesis. | - |
| Summary measures | 13 | State the principal summary measures (e.g., risk ratio, difference in means). | - |
| Synthesis of results | 14 | Describe the methods of handling data and combining results of studies | 8 |
| Risk of bias across studies | 15 | Specify any assessment of risk of bias that may affect the cumulative evidence (e.g., publication bias, selective reporting within studies). | - |
| Additional analyses | 16 | Describe methods of additional analyses (e.g., sensitivity or subgroup analyses, meta-regression), if done, indicating which were pre-specified. | - |
| **RESULTS** | | |  |
| Study selection | 17 | Give numbers of studies screened, assessed for eligibility, and included in the review, with reasons for exclusions at each stage, ideally with a flow diagram. | 9 |
| Study characteristics | 18 | For each study, present characteristics for which data were extracted (e.g., study size, PICOS, follow-up period) and provide the citations. | 11 |
| Risk of bias within studies | 19 | Present data on risk of bias of each study and, if available, any outcome level assessment (see item 12). | - |
| Results of individual studies | 20 | For all outcomes considered (benefits or harms), present, for each study: (a) simple summary data for each intervention group (b) effect estimates and confidence intervals, ideally with a forest plot. | 12 |
| Synthesis of results | 21 | Present results of each meta-analysis done, including confidence intervals and measures of consistency. | 12 |
| Risk of bias across studies | 22 | Present results of any assessment of risk of bias across studies (see Item 15). | - |
| Additional analysis | 23 | Give results of additional analyses, if done (e.g., sensitivity or subgroup analyses, meta-regression [see Item 16]). | - |
| **DISCUSSION** | | |  |
| Summary of evidence | 24 | Summarize the main findings including the strength of evidence for each main outcome; consider their relevance to key groups (e.g., healthcare providers, users, and policy makers). | 22-26 |
| Limitations | 25 | Discuss limitations at study and outcome level (e.g., risk of bias), and at review-level (e.g., incomplete retrieval of identified research, reporting bias). | 26-27 |
| Conclusions | 26 | Provide a general interpretation of the results in the context of other evidence, and implications for future research. | 28 |
| **FUNDING** | | |  |
| Funding | 27 | Describe sources of funding for the systematic review and other support (e.g., supply of data); role of funders for the systematic review. | 29 |

**Table A2** Literature search strategy **(**MEDLINE 1946 to July 2024)

| - - 1. Pregnancy/ |
| --- |
| - - 1. Pregnan*.tw. |
| - - 1. Gravidity/ |
| - - 1. Gravid*.tw. |
| - - 1. Gestation*.tw. |
| - - 1. Pregnant Women/ |
| - - 1. Pregnant wom#n.tw. |
| - - 1. Prepregnan*.tw. |
| - - 1. Preconception*.tw. |
| - - 1. (child adj3 bearing).tw. |
| - - 1. Childbearing.tw. |
| - - 1. Matern*.tw. |
| - - 1. or/1-12 |
| - - 1. Weight Gain/ph [Physiology] |
| - - 1. Weight gain*.tw. |
| - - 1. Weight Loss/ph [Physiology] |
| - - 1. Weight loss*.tw. |
| - - 1. Weight change*.tw. |
| - - 1. Obesity/dh, me, ph, pc, px, th [Diet Therapy, Metabolism, Physiology, Prevention & Control, Psychology, Therapy] |
| - - 1. Obes*.tw. |
| - - 1. Adiposity/ph |
| - - 1. Adipos*.tw. |
| - - 1. Overweight/dh, me, ph, pc, px, th [Diet Therapy, Metabolism, Physiology, Prevention & Control, Psychology, Therapy] |
| - - 1. Overweight*.tw. |
| - - 1. Body Mass Index/ |
| - - 1. Bmi.tw. |
| - - 1. or/14-26 |
| - - 1. Exp Randomized Controlled Trial/ |
| - - 1. "randomized controlled trial".pt. |
| - - 1. "controlled clinical trial".pt. |
| - - 1. (random$ or placebo$).tw,sh. |
| - - 1. ((singl$ or double$ or triple$ or treble$) and (blind$ or mask$)).tw,sh. |
| - - 1. Single-blind method/ |
| - - 1. Double-blind method/ |
| - - 1. or/28-34 |
| - - 1. 13 and 27 and 35 |
| - - 1. Exp Animals/ |
| - - 1. (rat$ or mouse or mice or hamster$ or animal$ or dog$ or cat$ or bovine or sheep or lamb$).af. |
| - - 1. 37 or 38 |
| - - 1. Humans/ |
| - - 1. Human$.tw,ot,kf. |
| - - 1. 40 or 41 |
| - - 1. 39 not 42 |
| - - 1. 36 not 43 |

**Table A3** Literature search strategy **(**EMBASE 1946 to July, 2024)

| 1. Pregnancy/ |
| --- |
| 1. Pregnan*.tw. |
| 1. Gravidity/ |
| 1. Gravid*.tw. |
| 1. Gestation*.tw. |
| 1. Pregnant Women/ |
| 1. Pregnant wom#n.tw. |
| 1. Prepregnan/ |
| 1. Prepregnan*.tw. |
| 1. Preconception/ |
| 1. Preconception*.tw. |
| 1. (child adj3 bearing).tw. |
| 1. Childbearing.tw. |
| 1. Matern*.tw. |
| 1. or/1-14 |
| 1. Weight Gain/ |
| 1. Weight gain*.tw. |
| 1. Weight Loss/ |
| 1. Weight loss*.tw. |
| 1. Weight change*.tw. |
| 1. Exp Obesity/ |
| 1. Obes*.tw. |
| 1. Adipos*.tw. |
| 1. Overweight*.tw. |
| 1. Body Mass Index/ |
| 1. Bmi.tw. |
| 1. or/16-26 |
| 1. Clinical trial/ |
| 1. Randomized controlled trial/ |
| 1. Controlled clinical trial/ |
| 1. Multicenter study/ |
| 1. Phase 3 clinical trial/ |
| 1. Phase 4 clinical trial/ |
| 1. Exp randomization/ |
| 1. Single blind procedure/ |
| 1. Double blind procedure/ |
| 1. Crossover procedure/ |
| 1. Placebo/ |
| 1. Randomi?ed controlled trial$.tw. |
| 1. RCT.tw. |
| 1. (random$ adj2 allocat$).tw. |
| 1. Single blind$.tw. |
| 1. Double blind$.tw. |
| 1. ((treble or triple) adj blind$).tw. |
| 1. Placebo$.tw. |
| 1. Prospective Study/ |
| 1. or/28-46 |
| 1. Exp animal/ not human.sh. |
| 1. 47 not 48 |
| 1. 15 and 27 and 49 |

**Table A4** Literature search strategy (Cochrane to July, 2024)

| #1 Pregnan* |
| --- |
| #2 MeSH descriptor: [Pregnancy] explode all trees |
| #3 Gestation |
| #4 Pregnant women |
| #5 MeSH descriptor: [Pregnant Women] explode all trees |
| #6 MeSH descriptor: [Gravidity] explode all trees |
| #7 Prepregnan* |
| #8 Preconception or periconception |
| #9 Gravidity |
| #10 #1 OR #2 OR #3 OR #4 OR #5 OR #6 OR #7 OR #8 OR #9 |
| #11 Weight gain |
| #12 Body mass index |
| #13 MeSH descriptor: [Body Mass Index] explode all trees |
| #14 BMI |
| #15 Obes* |
| #16 MeSH descriptor: [Obesity] explode all trees |
| #17 Overweight |
| #18 MeSH descriptor: [Overweight] explode all trees |
| #19 #11 OR #12 OR #13 OR #14 OR #15 OR #16 OR #17 OR #18 |
| #20 Diet |
| #21 MeSH descriptor: [Diet] explode all trees |
| #22 Lifestyle |
| #23 MeSH descriptor: [Life Style] explode all trees |
| #24 Exercise |
| #25 MeSH descriptor: [Exercise] explode all trees |
| #26 Physical activity |
| #27 Pharmacotherapy |
| #28 Metformin |
| #29 MeSH descriptor: [Metformin] explode all trees |
| #30 Intervention |
| #31 #20 OR #21 OR #22 OR #23 OR #24 OR #25 OR #26 OR #27 OR #28 OR #29 OR #30 |
| #32 MeSH descriptor: [Animals] explode all trees |
| #33 MeSH descriptor: [Humans] explode all trees |
| #34 #32 NOT #33 |
| #35 #10 AND #19 AND #31 |
| #36 #35 NOT 34 with Cochrane Library publication date Between Feb 2022 and Feb 2023, in Trials |

**Table A5** Summary of PICOS criteria for the inclusion of studies

| Parameter | Description |
| --- | --- |
| Population | Preconception or pregnant women at higher risk of GDM, identified using risk factors (e.g., overweight/obesity, raised lipids, elevated glucose concentration, insulin resistance, maternal age, high-risk ethnicity, previous macrosomic infant, previous GDM, PCOS, metabolic syndrome, hypertension, family history of GDM or diabetes, use of a risk tool) |
| Intervention | Behavioural (diet/ PA/ diet and PA) and/or supplements and/or pharmacological intervention |
| Comparison | No intervention, standard care, or placebo |
| Outcome | GDM as a primary or secondary outcome |
| Study design | Randomised controlled trials with a qualitative component  English language only  No restriction on publication date |

Abbreviations: GDM= gestational diabetes mellitus; PA= physical activity; PCOS= polycystic ovary syndrome.

**Table A6** Critical Appraisal Skills Programme (CASP) qualitative checklist table

| CASP Question | Amaefule *et al.,* 2022)^1^ | Chang *et al.,* 2023^2^ | Michalopolou *et al.,* 2023^3^ | Poston *et al.,* 2013^4^ |
| --- | --- | --- | --- | --- |
| Appropriate data collection method? | YES | YES | YES | YES |
| Appropriate methodology? | YES | YES | YES | YES |
| Appropriate research design? | YES | YES | YES | YES |
| Appropriate recruitment strategy? | YES | YES | YES | YES |
| Clear relevant aim? | YES | YES | YES | YES |
| Clear statement of finding? | YES | CAN’T TELL | YES | YES |
| Ethical issues been considered? | YES | YES | YES | YES |
| Reflexivity discussed? | YES | CAN’T TELL | YES | YES |
| Sufficiently rigorous data analysis methods? | CAN’T TELL | CAN’T TELL | YES | YES |
| Valuable research question? | YES | YES | YES | YES |

**Table A7** Included study characteristics

| Reference | Country | Aims | Age (years) Mean (SD) | BMI (kg/m^2^) Mean (SD) | Education | Ethnicity | Sample size |
| --- | --- | --- | --- | --- | --- | --- | --- |
| Amaefule *et al.,* 2022^1^ | UK | To examine the feasibility and acceptability of conducting a randomised trial on the effects of myo-inositol in preventing gestational diabetes in high-risk pregnant women | Not reported | Not reported | Higher Education n= 12  Secondary education n= 3 | Middle Eastern: n= 3  South Asian: n= 5  White European: n= 6  Black African/ Caribbean: n= 1 | n= 28 observations  n= 15 interviews |
| Chang *et al.*, 2023 ^2^ | USA | To evaluate the feasibility of recruitment, retention, and intervention acceptability of a pilot lifestyle behaviour intervention that incorporated Hope theory and goal-oriented episodic future thinking to help overweight or obese pregnant participants prevent excessive gestational weight gain | Not reported | Not reported | Not reported | Not reported | n= 19 interviews |
| Michalopolou *et al.*, 2023^3^ | UK | To test the feasibility and acceptability of a reduced-carbohydrate diet program aimed at reducing the risk of gestational diabetes | Not reported | Not reported | Not reported | Not reported | n= 16 interviews |
| Poston *et al.*, 2013 ^4^ | UK | To determine if a) a complex intervention in obese pregnant women leads to anticipated changes in diet and physical activity behaviours, and b) to refine the intervention protocol through process evaluation of intervention fidelity | 29.6 (4.9) | 37.6 (4.6) | Not reported | White: n= 8  Black: n= 12  Other: n= 1 | n = 9 interviews with intervention arm  n = 12 interviews with control arm |

*Included study characteristics relate to the qualitative component of the study only.

**Table A8** Characteristics of interventions of included studies

| Reference | Intervention | Timepoint measuring acceptability | Outcomes | Data collection/ method |
| --- | --- | --- | --- | --- |
| Amaefule *et al.,* 2022^1^ | **Who provided the intervention:** Researchers  **Method of intervention delivery:** In person or by phone/App (technology)  **Study setting:** The trial was conducted in five inner city maternity units including Barts Health Trust (The Royal London Hospital, Whipps Cross University Hospital and Newham University Hospital), St George’s University Hospitals NHS Foundation Trust, and Manchester University Hospital NHS Foundation Trust (Manchester Royal Infirmary)  **Intervention duration:** From 12^+0^to 15^+6^ weeks’ gestation until delivery. Participants were followed-up in person or by phone at 20, 28 and 36 weeks’ gestation and at delivery.  **Dose/Frequency:**  I: 2 g of myo-inositol with 200 µg folic acid, two times a day in a powder form mixed in water.  C: Powder of Xylitol filler with 200 µg folic acid to be taken twice a day | Prior to intervention during recruitment and post intervention | Acceptability of the interventions   - Awareness of GDM - Extra support and antenatal care - Natural pharmacological supplement   Adherence to the intervention   - The intervention content and delivery - Inter-intra personal behaviours | Participant observations  Semi-structured interviews |
| Chang *et al.,* 2023 ^2^ | **Who provided the intervention:** Health coach  **Method of intervention delivery:** Web intervention module with 2 parts (using Qualtrics software, a total of 20 modules, 30 min/module)  Individual health coaching through Zoom (a total of 10 sessions, 45 min/session)  **Study setting:** Clinicians from 5 prenatal care clinics affiliated with Ohio State University Wexner Medical Center referred participants to this study.  **Intervention duration:** From < 17 weeks gestation until 35-37 weeks gestation weeks. The participants were followed up at 24-27 weeks gestation and 35-37 weeks gestation (online)  **Dose/Frequency:**  I: A weekly web intervention module with 2 parts (I and II) and individual health coaching sessions (10 sessions). The interventions focused on three main topics: stress and emotion management, healthy eating, and physical activity. Web intervention with 20 sessions: six modules include stress and emotion management, 10 modules cover healthy eating, and 4 modules cover physical activity. The Web part I (25 minutes) intervention included written text only. Part I encouraged participants to manage stress and emotion by asking them to picture a goal-related event selected. The part II (5 minutes) included goal progress evaluation. Participants conducted 10 health coaching sessions (45 minutes per session) weekly (weeks 1-4) followed by every other week (weeks 6-12) and monthly (weeks 16-20). The health coaching helped to reinforce/modify information from the web intervention.  C: Usual prenatal care | Post intervention | Acceptability of intervention   - Extra support and antenatal care   Adherence to the interventions   - The intervention content and delivery - Technology based interventions - Provision of written materials - The personalised approach of the health coach - Frequency of intervention sessions | Semi-structured interviews |
| Michalopolou *et al.,* 2023^3^ | **Who provided the intervention:** Research dietitian/Healthcare professionals  **Method of delivery:** In person and by phone  **Study setting:** The Women's Centre at Oxford University Hospitals NHS Foundation Trust  **Intervention duration:** From <20 weeks' gestation until delivery. Brief telephone support sessions at around : 2, 4, 8 , 12,16 and 20 weeks from baseline  **Dose/Frequency:**  I: Aimed 130–150 g total carbohydrate/day, with no specific advice to change protein or fat intake, for approximately 6 months. Dietary advice focused on refraining sugary foods and drinks, reducing portions of starchy carbohydrates and swapping refined starchy carbohydrates with unrefined varieties, and sugary foods/drinks with low/no-sugar alternatives.  C: Routine antenatal care | Post intervention | Acceptability of intervention   - Awareness of GDM - Extra support and antenatal care   Adherence to interventions   - The intervention content and delivery - Smaller group sessions - Provision of written materials - Social support from family, partners and healthcare providers - Competing priorities - Psycho-social factors   Perceived Changes in Knowledge, Attitude and Behaviour | Semi-structured interviews |
| Poston *et al.,* 2013 ^4^ | **Who provided the intervention:** Health trainer  **Method of delivery:** In person  **Study setting:** In four UK study centres: The Southern General Hospital and Princess Royal Maternity Hospital (Glasgow), The Royal Victoria Infirmary (Newcastle), Guy’s and St Thomas’ NHS Foundation Trust (London) and King’s College Hospital Foundation Trust (London).  **Intervention duration:** From approximately 19 weeks’ gestation until 27^+0^ and 28^+6^ weeks’ gestation  8 consecutive weeks from approximately 19 weeks’ gestation  Participants were followed up at 27^+0^-28^+6^ weeks’ gestation  34^+0^-35^+6^ weeks’ gestation  **Dose/Frequency:**  I: Individualised behavioural intervention (face-to-face/telephone/email group or individual 8 sessions; 1-hr) delivered by a health trainer. The dietary component of the intervention focused on promoting healthy eating without restricting energy intake. The advice was tailored based on the woman’s habitual diet and cultural preference. Advice focused on swapping carbohydrate-rich foods with medium-to-high GI for foods with a lower GI and reducing saturated fat intake. The physical activity component aimed to promote incremental increases in walking at a moderate intensity tailored to pre-existing activities. The intervention covered the following: self-monitoring, problem-solving, enlisting social support, and providing opportunities for social comparison. A handbook, a DVD, a pedometer, and a logbook were provided to women.  C: Standard antenatal care is in line with local practice and includes general information regarding healthy eating and the benefits of physical activity. | Post intervention | Acceptability of intervention   - Awareness of GDM - Extra support and antenatal care   Adherence to interventions   - The intervention content and delivery - Smaller group sessions - Social support from family, partners and healthcare providers - Provision of written materials and tools to record and measure progress- handbook or logbook - Provision of pedometer - Dietary advice provided by the health trainer - Competing priorities   Perceived Changes in Knowledge, Attitude and Behaviour | Semi-structured interviews |

**References**

1. Amaefule CE, Drymoussi Z, Carreras FJG, et al. Myo-inositol nutritional supplement for prevention of gestational diabetes (EMmY): a randomised, placebo-controlled, double-blind pilot trial with nested qualitative study. BMJ Open. 2022;12(3). doi:10.1136/bmjopen-2021-050110
2. Chang MW, Tan A, Schaffir J, et al. A pilot lifestyle behavior intervention for overweight or obese pregnant women: results and process evaluation. J Pediatr Perinatol Child Health. 2023;7(1). doi:10.26502/jppch.74050139
3. Michalopoulou M, Jebb SA, MacKillop LH, et al. REduced-Carbohydrate intervention for managing obesity and reduction of gestational diabetes (RECORD): a randomized controlled feasibility trial. Diabetes Obes Metab. 2024;26(4):1407–20. doi:10.1111/dom.15442
4. Poston L, Briley AL, Barr S, et al. Developing a complex intervention for diet and activity behaviour change in obese pregnant women (the UPBEAT trial); assessment of behavioural change and process evaluation in a pilot randomised controlled trial. BMC Pregnancy Childbirth. 2013;13. doi:10.1186/1471-2393-13-148
